# Sustained Resolution of Multifocal Low-Grade Dysplasia in Ulcerative Colitis

**DOI:** 10.14309/crj.0000000000000178

**Published:** 2019-08-21

**Authors:** Andrew Canakis, Peter Dellatore, Matthew Josephson, Justin Canakis, Zainab Alruwaii, Mark Lazarev, Steven R. Brant

**Affiliations:** 1 Department of Medicine, Boston Medical Center, Boston University School of Medicine, Boston, MA; 2 Crohns and Colitis Center of New Jersey, Division of Gastroenterology and Hepatology, Department of Medicine, Rutgers Robert Wood Johnson Medical School, New Brunswick, NJ; 3 Philadelphia College of Osteopathic Medicine, Philadelphia, PA; 4 Department of Pathology, Johns Hopkins University School of Medicine, Baltimore, MD; 5 The Harvey M. and Lyn P. Meyerhoff Inflammatory Bowel Disease Center, Department of Medicine, Johns Hopkins University School of Medicine, Baltimore, MD

## Abstract

In inflammatory bowel disease, prolonged disease duration, pancolitis, histological inflammation, and subsequent dysplasia are associated with an increased risk for colorectal cancer. Recommendations regarding treatment of low-grade dysplasia (LGD) indicate an individualized approach between colectomy and surveillance. We present a unique case of a patient with ulcerative colitis who had multifocal LGD on 2 consecutive colonoscopies. However, after 10 years and 16 surveillance colonoscopies, she had no further evidence of dysplasia. This appears to be the first case of proven, permanently resolved multifocal LGD in inflammatory bowel disease that challenges our understanding of the natural history of LGD.

## INTRODUCTION

Because of long-standing colonic inflammation, patients with inflammatory bowel disease (IBD) are at an increased risk for developing colorectal cancer (CRC). Studies have estimated that CRC incidence in patients with IBD is 6 times higher than that in the general population.^1,2^ Risk factors include extent and chronicity of disease, familial history of CRC, primary sclerosing cholangitis, and severe disease with active endoscopic and histological signs of inflammation.^3–5^ A meta-analysis of 116 studies found that the probability of CRC in patients with ulcerative colitis increased with disease duration; the incidence of CRC was 1.6% at 10 years, 8.3% at 20 years, and 18.4% by 30 years of disease duration.^6^

The assumed, stepwise progression of carcinogenesis in IBD is thought to be from chronic inflammation that leads to dysplasia (low then high grade) and then adenocarcinoma.^5–9^ Although current guidelines recommend surveillance colonoscopy every 2 years, beginning after 8 years of disease, it is noteworthy that these associated risk factors are not equivalent in all patients, and dysplasia progression cannot be predicted.^1,9^ For instance, patients undergoing routine surveillance may develop cancer without previous histological diagnosis of dysplasia, whereas others may progress directly from low-grade dysplasia (LGD) to carcinoma.^9^ The role of surveillance protocols in lowering the incidence of CRC remains unclear. This uncertainty is compounded by the fact that surveillance protocols and standardized definitions of dysplastic lesions have changed over time—thus conflicting previous epidemiological studies.^10^

High-grade dysplasia (HGD) generally warrants surgery, especially because coexisting adenocarcinoma can be found in 42% of colons at resection.^11^ However, approaches toward LGD remain uncertain because its natural progression is inadequately defined. Current recommendations regarding decisions to opt for colectomy vs intensive surveillance are for an individualized approach, with a strong emphasis on educating patients on the risks and benefits of different choices.

## CASE REPORT

**Figure 1.**
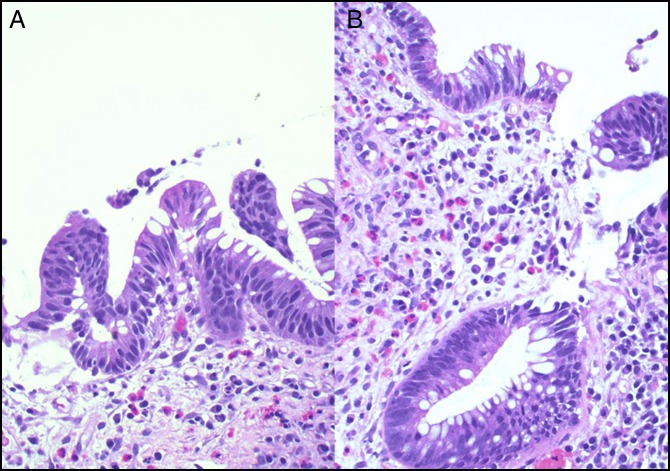
(A and B) Hematoxylin and eosin stain of the colonic mucosa with low-grade dysplasia showing nuclear hyperchromasia, enlargement, elongation (×400).

**Figure 2.**
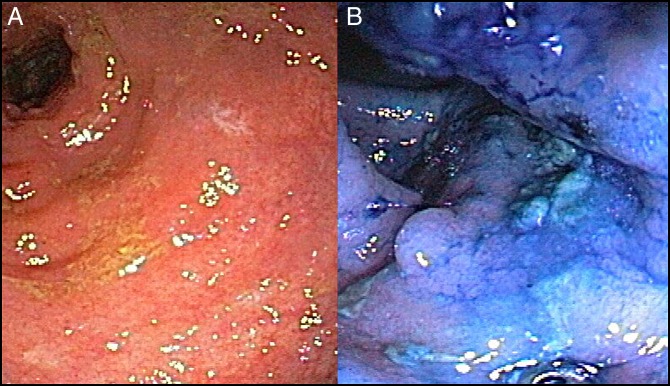
(A) Colonoscopy in 2008 showing the rectum, and (B) chromoendoscopy in 2008 showing the sigmoid colon where multifocal low-grade dysplasia was found.

A 46-year-old woman had been followed up for pan-ulcerative colitis since 2002. She was transferred to our care in 2007. She had no other illnesses, extraintestinal manifestations of IBD, or primary sclerosing cholangitis. She was diagnosed at the age of 30 years, initially managed with mesalamine and hydrocortisone enemas, but after 4 years, she required azathioprine to control the disease. In 2008, 2 years after her previous flare, random rectal biopsies showed multifocal LGD (confirmed at gastrointestinal pathology conference) in the setting of inactive chronic inflammation with left-sided colitis and proctitis (Figures 1 and 2). Repeat sigmoid and rectal biopsies 6 months later confirmed multifocal LGD. The 2 initial colonoscopies were consistent with Mayo 0 and Mayo 1, respectively. No polypoid or mass-like lesions were observed, and no endoscopic resections were performed on either colonoscopy. Despite a strong recommendation to undergo total proctocolectomy, the patient opted for intensive semiannual surveillance with chromoendoscopy.

**Figure 3.**
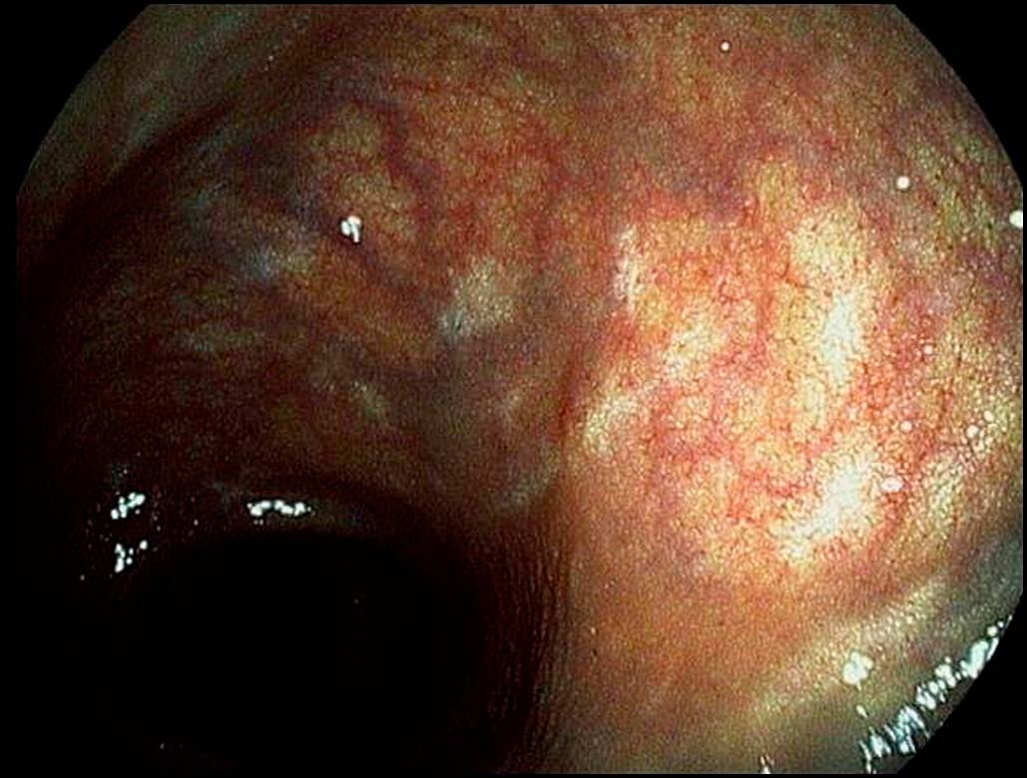
Colonoscopy in 2018 showing healing at the rectum.

The patient underwent colonoscopies (by the same 2 gastroenterologists) every 6 months for 5 years and then yearly for the next 5 years with no further signs of dysplasia. She exhibited endoscopic Mayo scores of 0 on all subsequent colonoscopies, except when she had a flare in 2015 (Mayo 2 at the time). She remained on mesalamine and azathioprine. Six of the 16 colonoscopies were with methylene blue chromoendoscopy, and all colonoscopies after 2012 were with high-definition endoscopes. All colonoscopies were conducted with 4 quadrant biopsies every 10 cm, with additional biopsies in the sigmoid and rectum, and all biopsies were reviewed by the faculty of the Division of Gastrointestinal and Liver Pathology, which has an established consultation service for evaluating IBD-related dysplasia. Of note, the patient only had one colitis flare (in 2015) since the diagnosis of LGD. It required 8 weeks of prednisone; however, colonoscopies continued to show no evidence of LGD (Figure 3). Interestingly, she is an active runner and has run multiple marathons before and after her IBD diagnosis.

## DISCUSSION

Current recommendations regarding treatment of LGD indicate an individualized approach between colectomy and surveillance. The estimated risk of progression from LGD to HGD or CRC is varied, and before this case, dysplasia had not been shown to completely resolve. In fact, a meta-analysis demonstrated that once LGD is diagnosed, the risk of cancer or any advance lesion was 9- and 12-fold, respectively.^12^ Mount Sinai Hospital reported a 5-year rate of progression of LGD to HGD or CRC of 45%.^13^ Two additional studies examined the specimens of patients who underwent colectomy after diagnosis of LGD and reported a 27% and 19% unexpected discovery of HGD and CRC.^11,14^ These reports have led many practitioners to advocate for total proctocolectomy in LGD.

Despite this evidence, there still remains substantial variability in the literature, and many clinicians opt for intensive surveillance strategies because of a multitude of studies that reported lower rates of CRC progression.^8^ A prospective Swedish study found no progression to CRC in 60 patients with long-standing IBD with LGD, whereas another retrospective study did not find a statistically significant difference in CRC progression to reliable justify colectomy.^15,16^ It is well established that when HGD (not confined to a resectable lesion) is confirmed, colectomy is mandatory. Regarding LGD, if colectomy is deferred, then it is critical to educate the patient on the risk of interval cancer despite an intensive surveillance program.

Why the dysplasia resolved is unclear. One area to explore may be the effect of her long distance running lifestyle. Perhaps her daily exercise induced apoptosis of dysplastic colonic cells, parallel to the effects reported of exercise reducing lung cancer progression in mouse models.^17^ It is well established that routine exercise can reduce colon cancer mortality.^18,19^

We conclude from this case report that established, multifocal LGD can resolve. We note however that although this case increases our understanding of the natural history of LGD, until dysplasia progression is better understood, both IBD specialists (M.L. and S.R.B.) who contributed to this report (and cared for the patient) continue to advise patients with multifocal LGD to undergo colectomy as the best option, given risks of progression and that CRC may not be detected on follow-up surveillance. Rather, this finding should spur more research into the natural history of LGD with a goal to identify biomarkers, tissue (eg, molecular, histologic) characteristics, and treatment options that may result in similarly favorable outcomes.

## DISCLOSURES

Author contributions: A. Canakis reviewed the literature, researched the case reports, and wrote the manuscript. P. Dellatore, J. Canakis, and M. Josephson researched the case report. Z. Alruwaii provided the pathology data and photos. M. Lazarev and SR Brant provided case information, literature review, endoscopy photos, and critical editing of the manuscript. SR Brant is the article guarantor. All authors reviewed the final manuscript.

Financial disclosure: None to report.

Previous presentation: This case report was presented in part at the 2018 ACG Annual Meeting; October 5-10, 2018; Philadelphia, PA.

Informed consent was obtained for this case report.
